# Psychosocial and occupational correlates of burnout among nurses from selected hospitals in three countries in Central Europe: a cross-sectional study

**DOI:** 10.3389/fpubh.2026.1804266

**Published:** 2026-06-09

**Authors:** Krystyna Kowalczuk, Katarzyna Tomaszewska, Helena Kadučáková, Anna Krátká, Erika Durejova, Bożena Majchrowicz, Marek Sobolewski, Justyna M. Hermanowicz

**Affiliations:** 1 Department of Integrated Medical Care, Medical University of Bialystok, Białystok, Poland; 2 Department of Nursing, Institute of Health Protection, The Bronislaw Markiewicz State Higher School of Technology and Economics, Jaroslaw, Poland; 3 Department of Nursing, Faculty of Health Sciences, Catholic University in Ružomberok, Ružomberok, Slovakia; 4 Department of Health Care Sciences, Faculty of Humanities, Tomas Bata University in Zlin, Zlín, Czechia; 5 Ustredna Vojenska Nemocnica Ružomberok, Ružomberok, Slovakia; 6 Department of Nursing, Institute of Health Protection, State Academy of Applied Sciences, Przemyśl, Poland; 7 Faculty of Management, Rzeszow University of Technology, Rzeszów, Poland; 8 Department of Pharmacodynamics, Medical University of Bialystok, Białystok, Poland

**Keywords:** demographic factors, nurses, occupational burnout, occupational correlates, perceived stress, public health

## Abstract

**Introduction:**

Occupational burnout among nurses constitutes an important public health issue due to its impact on workforce sustainability and quality of care. Although stress is considered a key correlate of burnout, evidence regarding associations between psychosocial, demographic, and occupational factors and burnout in Central European settings remains limited.

**Methods:**

A cross-sectional study was conducted between March 2024 and March 2025 among 610 nurses employed in selected hospitals in Central Europe. Occupational burnout was assessed using the Link Burnout Questionnaire (LBQ), and perceived stress was measured with the Perceived Stress Scale (PSS-10). Associations between burnout, stress, and demographic and occupational variables were examined using nonparametric methods and Spearman’s rank correlations. Multivariable linear regression models were applied to assess variables associated with burnout severity.

**Results:**

Burnout levels were moderate, with the highest scores observed for psychophysical exhaustion and disengagement from patient relationships. More than one-third of nurses reported high perceived stress. Perceived stress showed significant weak-to-moderate correlations with all burnout dimensions and remained the strongest correlate in multivariable analyses, while demographic and occupational variables showed generally weaker associations with burnout dimensions.

**Conclusion:**

Occupational burnout among nurses was more strongly associated with perceived stress than with demographic or occupational characteristics. Public health interventions should prioritize reducing psychosocial stressors and improving working conditions in nursing environments.

## 1 Introduction

Occupational burnout represents a significant issue in occupational health among healthcare workers and has attracted increasing attention from a public health perspective. This phenomenon is particularly evident among nurses, whose work involves high emotional demands, substantial responsibility, and intensive, long-term contact with patients (1, 2). In the context of nursing workforce shortages and growing population health needs, burnout among nurses constitutes not only an individual but also a systemic challenge.

The concept of occupational burnout was first described by Freudenberger in the 1970s as a state of physical and psychological exhaustion, reduced motivation, and loss of engagement among individuals working in helping professions (3). The concept was subsequently developed by Maslach and Jackson (4), who proposed a multidimensional model of burnout encompassing emotional exhaustion, depersonalization, and reduced personal accomplishment. Contemporary approaches conceptualize occupational burnout as a psychological syndrome emerging in response to chronic psychosocial stressors in the workplace. According to the World Health Organization’s ICD-11 classification, burnout is defined as an occupational phenomenon related to work functioning rather than a medical condition (5).

The significance of burnout in nursing extends beyond individual well-being and has important implications for healthcare system functioning. Higher levels of burnout have been associated with lower job satisfaction, impaired teamwork, and reduced quality of patient care (6). Moreover, the literature highlights a strong relationship between burnout and intentions to leave the profession, as well as increased staff turnover, which represents a major public health concern in the context of nursing shortages (7).

Burnout is also closely linked to nurses’ mental and physical health. Research indicates that burnout frequently co-occurs with depressive and anxiety symptoms, sleep disturbances, chronic fatigue, and somatic complaints, including musculoskeletal and cardiovascular problems (8, 9). From a public health perspective, occupational burnout may therefore be viewed as a factor contributing to the deterioration of healthcare workers’ health and increasing the burden on healthcare systems.

One of the key psychosocial phenomena examined in the context of burnout is stress. In the classic transactional model, stress is defined as a relationship between the individual and the environment that is appraised as exceeding available resources and threatening well-being (10). In nursing practice, sources of stress include organizational factors such as workload, time pressure, and responsibility for patients’ health, as well as emotional burdens resulting from exposure to suffering, illness, and death.

Previous studies have demonstrated statistically significant associations between perceived stress and burnout severity among nurses (11, 12). However, these relationships reflect co-occurrence, and their interpretation should consider the limitations of cross-sectional designs, which do not allow causal inference. From a public health perspective, identifying correlates of burnout—including stress and demographic and occupational factors—is essential for designing preventive and organizational interventions. In the present study, perceived stress was treated as the primary psychosocial factor analyzed in relation to occupational burnout.

Despite the growing body of research on burnout in nursing, data concerning nurses working in Central European countries remain limited. Many studies focus on single countries or specific work environments, highlighting the need for further research encompassing diverse occupational contexts within this region.

The aim of this cross-sectional study was to examine psychosocial, demographic, and occupational correlates of occupational burnout among nurses employed in selected hospitals in Central European countries. Specifically, the following research questions were addressed:

1. Is the level of perceived stress significantly associated with the severity of occupational burnout and its individual dimensions among nurses?
2. Are nurses’ demographic characteristics (age, marital status, having children) significantly associated with the level of occupational burnout?
3. Are nurses’ occupational characteristics (length of service, level of education, type of ward) significantly associated with the level of occupational burnout?
4. Which of the analyzed factors remain independently associated with occupational burnout in multivariable analysis?

## 2 Materials and methods

### 2.1 Study design and setting

A cross-sectional study was conducted between March 2024 and March 2025 in three hospitals located in Central European countries: the Czech Republic, Poland, and Slovakia. In each country, the study was carried out in a single hospital—specifically in Zlín (Czech Republic), Białystok (Poland), and Ružomberok (Slovakia).

The selected hospitals were comparable in terms of size (number of beds), teaching status, and range of inpatient services. All three institutions are relatively large hospitals within their respective national healthcare systems and are university hospitals or closely affiliated with local medical universities. In each hospital, all major inpatient wards were included in the study, namely internal medicine wards, surgical wards, and emergency departments, in order to ensure broad representation of clinical work environments.

The study protocol was approved by the appropriate authorities. In Zlín and Ružomberok, approval was obtained from hospital administrations, while in Białystok the study was approved by the Bioethics Committee of the Medical University of Białystok. All procedures involving human participants were conducted in accordance with international ethical standards, including voluntary participation, informed consent, anonymity, confidentiality, avoidance of harm, and transparent communication of study objectives and results.

### 2.2 Study group selection and data collection

The inclusion criterion was full-time hospital employment under a standard employment contract. Nurses working part-time or under employment arrangements other than a formal employment contract were excluded from participation.

In each participating hospital, 300 paper-based questionnaires were distributed to nurses who met the inclusion criteria and were present at work during the data collection period. The number of distributed questionnaires reflected organizational feasibility and the availability of eligible staff during data collection rather than the total number of nurses employed at each institution. Therefore, the distributed questionnaires should not be interpreted as covering the entire nursing workforce in the participating hospitals. Participation was voluntary and anonymous, and respondents were informed that they could withdraw from the study at any time without providing a reason. The study was based on a convenience sampling approach, as participation depended on nurses’ availability during the study period and their willingness to participate. Because participation was fully anonymous, no identifying information was collected and no follow-up with non-respondents was possible. Consequently, non-response bias could not be formally assessed. Nurses were asked to complete the questionnaires in their free time and return them in sealed envelopes to ensure confidentiality.

Of the 900 questionnaires distributed, 610 were returned correctly completed, yielding an overall response rate of 67.8%. Response rates differed by country: 81.6% in Poland, 71.3% in Slovakia, and 50.0% in the Czech Republic. The final study sample consisted of 610 licensed nurses employed in hospitals in Zlín, Czech Republic (*n* = 151), Białystok, Poland (*n* = 245), and Ružomberok, Slovakia (*n* = 214). Differences in sample size across countries resulted exclusively from variation in questionnaire return rates, as participation was voluntary and reasons for nonresponse were not assessed. Due to the anonymous study design, it was not possible to determine whether respondents differed systematically from non-respondents with respect to burnout, stress, or occupational characteristics. All demographic and occupational data were obtained via self-report, and no incentives were offered for participation.

### 2.3 Measures

#### 2.3.1 Occupational burnout

Occupational burnout was assessed using the Link Burnout Questionnaire (LBQ). The LBQ is a validated multidimensional instrument developed for professions involving intensive interpersonal engagement, particularly helping professions such as healthcare workers, teachers, and social care professionals (13, 14).

The instrument consists of 24 items rated on a six-point Likert scale and measures four dimensions of occupational burnout: (1) disappointment, (2) lack of professional effectiveness, (3) disengagement from relationships with patients, and (4) psychophysical exhaustion.

All four dimensions are negatively oriented, meaning that higher scores indicate higher levels of occupational burnout. Each subscale ranges from 6 to 36 points. A minimum score reflects a highly positive attitude toward professional work in the respective dimension, whereas a maximum score reflects a completely negative attitude.

The LBQ was selected because its conceptual framework emphasizes relational and emotional aspects of professional functioning that are particularly relevant in nursing practice, including deterioration of therapeutic engagement and disappointment related to helping professions. In addition, the instrument has previously been applied in studies conducted in healthcare settings in Central and Eastern Europe, supporting its contextual relevance for the present study population.

Previous studies have demonstrated satisfactory psychometric properties of the LBQ, including acceptable to good internal consistency of the subscales (Cronbach’s *α* typically ranging from approximately 0.68 to 0.85) as well as evidence supporting construct validity and multidimensional structure (15, 16).

Although more internationally widespread instruments for assessing burnout exist, particularly the Maslach Burnout Inventory (MBI), the LBQ was considered suitable for the present study because of its multidimensional approach and emphasis on relational aspects of burnout relevant to nursing care.

#### 2.3.2 Perceived stress

Perceived stress was measured using the Perceived Stress Scale (PSS-10) (17, 18). This instrument assesses the subjective appraisal of stress experienced during the previous month and consists of 10 items rated on a five-point Likert scale. Total scores range from 0 to 40 points, with higher scores indicating higher levels of perceived stress.

The PSS-10 is one of the most widely used instruments for assessing perceived stress and has demonstrated good reliability and validity across diverse cultural and occupational groups, including healthcare professionals (19, 20). Total scores range from 0 to 40 points, with higher scores indicating higher levels of perceived stress. Based on published normative data and the questionnaire authors’ recommendations, PSS-10 scores were categorized into three levels of perceived stress: low (<14 points), moderate (14–19 points), and high (≥20 points).

#### 2.3.3 Translation and linguistic adaptation of study instruments

Validated Polish versions of both instruments served as the basis for the study. Czech and Slovak versions were developed through forward translation performed by bilingual researchers affiliated with partner universities in the participating countries. The translated versions were subsequently reviewed collaboratively by the research team to ensure linguistic consistency and conceptual clarity.

The collaborating institutions maintain long-standing academic partnerships and regular staff exchange, which facilitated the harmonization of terminology and interpretation across language versions. However, a full formal cross-cultural adaptation procedure—such as forward–backward translation with independent translators, pilot testing, and psychometric equivalence assessment—was not conducted. Therefore, potential differences in cultural interpretation of some questionnaire items cannot be entirely excluded.

### 2.4 Statistical analysis

Statistical analyses were performed using Statistica 13.3 software. Distributional properties of the variables were assessed using descriptive statistics. As the LBQ burnout measures demonstrated non-normal distributions, nonparametric statistical methods were applied for analyses involving these variables. In contrast, the distribution of PSS-10 scores approximated normality sufficiently to justify the use of parametric methods; therefore, one-way analysis of variance (ANOVA) was used to compare mean perceived stress levels between countries.

Associations between perceived stress and occupational burnout were examined using Spearman’s rank correlation coefficient. For interpretative purposes, correlation coefficients ranging approximately from 0.30 to 0.50 were considered weak to moderate associations, according to commonly used guidelines for behavioral and health sciences research. Relationships between burnout dimensions and demographic and occupational variables were analyzed using nonparametric tests appropriate for the level of measurement of the variables, including the Kruskal–Wallis test.

To identify factors independently associated with occupational burnout in multivariable analysis, linear regression models were constructed. Separate models were developed for each of the four LBQ burnout dimensions. Diagnostic procedures were performed for all regression models, including assessment of residual distributions, homoscedasticity, linearity, and multicollinearity. Although some LBQ variables demonstrated skewed distributions, the residuals of the regression models showed no substantial deviations from normality or homoscedasticity. Additional analyses using logarithmically transformed burnout measures yielded comparable results. Psychosocial, demographic, and occupational variables that showed statistically significant associations in univariable analyses were entered into the regression models. Results are presented as regression coefficients with 95% confidence intervals and coefficients of determination (*R*^2^).

Although several dependent variables demonstrated non-normal distributions, assumptions of the regression models were additionally evaluated using post-hoc residual diagnostics, including assessment of residual distributions and residual-versus-fitted-value plots. These analyses did not indicate substantial violations of normality, linearity, or homoscedasticity assumptions. Additional analyses using log-transformed burnout measures produced substantively similar results, supporting the robustness of the reported models (see Table 1).

**Table 1:** Cronbach’s alpha coefficient for the individual psychometric measures (PSS-10 and LBQ) within each study sub-group.

Psychometric measure		Czech Rep.	Poland	Slovakia
PSS-10		0.730	0.824	0.659
LBQ	Disappointment	0.781	0.827	0.711
	Professional effectiveness	0.722	0.747	0.689
	Disengagement from patient relationships	0.683	0.688	0.649
	Psychophysical exhaustion	0.767	0.766	0.692

To facilitate interpretation, statistical significance was denoted as follows: *p* < 0.05 (*), *p* < 0.01 (**), *p* < 0.001 (***).

## 3 Results

### 3.1 Characteristics of the study groups

Women clearly predominated among the nurses (approximately 90% of all respondents, *p* < 0.001); therefore, sex was not included in further analyses. The age distribution of nurses was similar across the study groups (*p* > 0.05). In each group, married nurses predominated, with the smallest proportion observed in the Czech sample, which also showed the highest percentage of informal partnerships (28.7%). A similar proportion of nurses reported having children (approximately 50%, *p* < 0.001). In the Slovak sample, more than one-third of respondents did not answer this question.

The Polish sample included the highest proportion of nurses with master’s degrees (*p* < 0.001). Additionally, compared with the other samples, fewer nurses in Poland worked in internal medicine wards and more worked in surgical wards (*p* < 0.001). A comparative profile of the three samples is presented in Table 2.

**Table 2:** Comparative characteristics of the samples.

Characteristics/values		Country					
		Slovakia		Poland		Czech Rep.	
		*N*	%_↓_	*N*	%_↓_	*N*	%_↓_
Gender	Female	205	95.8%	219	89.4%	143	96.6%
	Male	9	4.2%	26	10.6%	5	3.4%
Age [years]	<28	33	15.4%	54	22.0%	36	24.5%
	28–33	16	7.5%	38	15.5%	13	8.8%
	34–39	24	11.2%	27	11.0%	13	8.8%
	40–45	52	24.3%	35	14.3%	29	19.7%
	46–51	37	17.3%	49	20.0%	31	21.1%
	52–57	33	15.4%	33	13.5%	22	15.0%
	>57	19	8.9%	9	3.7%	3	2.0%
Marital status	Partnership	46	21.5%	59	24.1%	41	28.7%
	Married	137	64.0%	163	66.5%	76	53.1%
	Single	31	14.5%	23	9.4%	26	18.2%
Children	Yes	104	48.6%	132	53.9%	84	55.6%
	No	36	16.8%	94	38.4%	51	33.8%
	No response	74	34.6%	19	7.8%	16	10.6%
Education	Nursing diploma	40	18.7%	34	13.9%	80	54.4%
	Bachelor’s degree	92	43.0%	85	34.7%	57	38.8%
	Master’s degree	82	38.3%	126	51.4%	10	6.8%
Years of experience	<6	57	26.6%	82	33.5%	45	31.5%
	6–11	43	20.1%	46	18.8%	24	16.8%
	12–17	30	14.0%	30	12.2%	21	14.7%
	18–23	35	16.4%	53	21.6%	31	21.7%
	>23	49	22.9%	34	13.9%	22	15.4%
Hospital ward	Internal medicine	115	59.3%	90	36.7%	78	59.5%
	Surgical	66	34.0%	102	41.6%	25	19.1%
	Emergency	13	6.7%	53	21.6%	28	21.4%

### 3.2 Occupational burnout (LBQ)

The results of the analysis of the occupational burnout questionnaire (LBQ) are presented in Table 3. The findings indicate a moderate level of occupational burnout in the study population, with clear differentiation between individual dimensions. Among the analyzed burnout dimensions, the highest scores were observed for disengagement from relationships with patients and psychophysical exhaustion, with particularly high psychophysical exhaustion noted in the Czech sample.

**Table 3:** Burnout level according to LBQ measures.

LBQ measure	Country	Burnout level					
		*N*	Mean (95% CI)	Median	Std. dev.	Min	Max
Disappointment (*p* = 0.3994)	Slovakia	209	14.5 (13.7–15.3)	14	5.9	6	34
	Poland	244	14.9 (14.1–15.7)	14	6.2	6	31
	Czech R.	150	15.5 (14.5; 16.5)	15	6.2	6	34
Professional effectiveness (*p* = 0.7686)	Slovakia	209	14.2 (13.6–14.9)	13	4.8	6	25
	Poland	244	14.5 (13.8–15.2)	14	5.3	6	29
	Czech R.	150	14.0 (13.2; 14.7)	13	4.5	6	26
Disengagement from patient relationships (*p* < 0.0001***)	Slovakia	209	17.0 (16.3–17.7)	17	4.8	7	31
	Poland	244	17.9 (17.2–18.6)	18	5.3	7	34
	Czech R.	150	19.7 (18.8; 20.6)	19	5.4	7	32
Psychophysical exhaustion (*p* = 0.0003***)	Slovakia	209	18.0 (17.3–18.7)	18	5.0	8	33
	Poland	244	18.1 (17.4–18.8)	18	5.5	7	34
	Czech R.	150	20.4 (19.4; 21.4)	20	6.1	6	35

*p*-test probability values calculated using the Kruskal–Wallis test. *p < 0.05, **p < 0.01, ***p < 0.001.

### 3.3 Perceived stress (PSS-10)

The results of the Perceived Stress Scale (PSS-10) analysis are presented in Table 4. The obtained values indicate a moderate level of perceived stress in the study population. No statistically significant differences were found in stress levels between Polish, Slovak, and Czech nurses (*p* > 0.05).

**Table 4:** Stress level according to the PSS-10 measure.

Country	Stress level PSS-10 (*p* = 0.0916)					
	*N*	Mean (95% CI)	Median	Std. dev.	Min	Max
Slovakia	208	17.5 (16.8–18.2)	17	5.0	4	31
Poland	243	17.1 (16.3–17.8)	17	6.2	2	34
Czech R.	151	18.3 (17.5–19.2)	19	5.4	3	33

*p*-test probability values calculated using ANOVA.

After classifying PSS-10 scores into a three-level scale, more than one-third of nurses were categorized as experiencing a high level of stress, as shown in Table 5.

**Table 5:** Stress levels by group.

Stress level	Country (*p* = 0.1188)			Total
	Slovakia	Poland	Czech Rep.	
Low	45 (21.6%)	69 (28.4%)	28 (18.5%)	142
Medium	84 (40.4%)	89 (36.6%)	55 (36.4%)	228
High	79 (38.0%)	85 (35.0%)	68 (45.0%)	232
Total	208	243	151	602

*p*-test probability values calculated using the chi-square test of independence.

### 3.4 Stress and occupational burnout

Higher levels of perceived stress (PSS-10) were statistically significantly correlated with higher levels of occupational burnout (LBQ) across all dimensions. The observed correlations were generally weak to moderate in strength, with Spearman’s rank correlation coefficients ranging approximately from 0.20 to 0.50. The strongest associations between burnout and stress were observed among Polish nurses, although for some measures similar correlations were also noted in the Czech sample.

Spearman’s rank correlation coefficients between burnout dimensions and perceived stress, along with their statistical significance, are presented in Table 6.

**Table 6:** Correlations between burnout and stress.

LBQ	Slovakia	Poland	Czech Rep.
	PSS-10		
Disappointment	0.20 (*p* = 0.0042**)	0.41 (*p* < 0.0001***)	0.40 (*p* < 0.0001***)
Professional effectiveness	0.41 (*p* < 0.0001***)	0.49 (*p* < 0.0001***)	0.32 (*p* = 0.0001***)
Disengagement from patient relationships	0.32 (*p* < 0.0001***)	0.48 (*p* < 0.0001***)	0.26 (*p* = 0.0013**)
Psychophysical exhaustion	0.37 (*p* < 0.0001***)	0.49 (*p* < 0.0001***)	0.48 (*p* < 0.0001***)

*p*-test probability value *p < 0.05, **p < 0.01, ***p < 0.001.

### 3.5 Social and occupational factors in relation to PSS-10 and LBQ

This section examined whether factors such as age, marital status, having children, length of service, education level, and ward type were associated with perceived stress and occupational burnout. Analyses were conducted separately for the Polish, Slovak, and Czech samples. Overall, statistically significant findings in the Czech sample were sporadic and of low effect size, without a consistent pattern across analyzed variables.

#### 3.5.1 Age and PSS-10 and LBQ

The results of correlations between age and PSS-10 and LBQ measures are presented in Table 7. In the Polish and Slovak samples, no statistically significant correlations were observed. In the Czech sample, two statistically significant but very weak correlations were found: perceived stress decreased with age (PSS-10; *r*_s_ = −0.19), as did psychophysical exhaustion (*r*_s_ = −0.23).

**Table 7:** Correlations of burnout and stress with age.

Psychometric measures	Slovakia	Poland	Czech Rep.
	Age [years]		
PSS-10	0.05 (*p* = 0.4893)	−0.11 (*p* = 0.0970)	−0.19 (*p* = 0.0190*)
Disappointment	0.02 (*p* = 0.7224)	0.09 (*p* = 0.1820)	−0.10 (*p* = 0.2245)
Professional effectiveness	0.03 (*p* = 0.7147)	−0.12 (*p* = 0.0697)	−0.11 (*p* = 0.1714)
Disengagement from patient relationships	−0.01 (*p* = 0.8412)	0.08 (*p* = 0.2324)	−0.10 (*p* = 0.2447)
Psychophysical exhaustion	0.11 (*p* = 0.1020)	0.05 (*p* = 0.4441)	−0.23 (*p* = 0.0051**)

*p*-test probability value *p < 0.05, **p < 0.01, ***p < 0.001.

#### 3.5.2 Marital status

Marital status did not significantly differentiate perceived stress levels or any of the LBQ burnout dimensions in any of the study groups (all *p*-values from the Kruskal–Wallis test were clearly above 0.05).

#### 3.5.3 Having children

In general, having children did not significantly differentiate levels of stress or occupational burnout. Only in the Czech sample did nurses with children report slightly lower perceived stress (17.7 vs. 19.5 points; *p* = 0.0439), lower disappointment (14.5 vs. 17.2 points; *p* = 0.0244), and lower lack of professional effectiveness (13.4 vs. 14.9 points; *p* = 0.0389).

#### 3.5.4 Education

Education level did not significantly differentiate perceived stress or any LBQ burnout dimensions.

#### 3.5.5 Length of service

Length of service did not differentiate stress levels in the Polish and Slovak samples. In the Czech group, a weak negative correlation was observed, indicating a slight decrease in stress with increasing length of service.

For burnout dimensions, statistically significant correlations were observed for disappointment in the Polish sample (*r*_s_ = 0.13; *p* = 0.0335), lack of professional effectiveness in the Czech sample (*r*_s_ = −0.18; *p* = 0.0284), and psychophysical exhaustion in the Slovak sample (*r*_s_ = 0.14; *p* = 0.0387). These relationships, although statistically significant, were very weak. Detailed results are presented in Table 8.

**Table 8:** Correlations of burnout and stress with length of service.

Psychometric measures	Slovakia	Poland	Czech Rep.
	Length of service [years]		
PSS-10	0.02 (*p* = 0.7891)	−0.09 (*p* = 0.1699)	−0.21 (*p* = 0.0108*)
Disappointment	0.06 (*p* = 0.3902)	0.13 (*p* = 0.0355*)	−0.02 (*p* = 0.8563)
Professional effectiveness	0.02 (*p* = 0.8096)	−0.07 (*p* = 0.2701)	−0.18 (*p* = 0.0284*)
Disengagement from patient relationships	0.05 (*p* = 0.4420)	0.11 (*p* = 0.0966)	0.00 (*p* = 0.9782)
Psychophysical exhaustion	0.14 (*p* = 0.0387*)	0.10 (*p* = 0.1152)	−0.14 (*p* = 0.0960)

*p*-test probability value *p < 0.05, **p < 0.01, ***p < 0.001.

#### 3.5.6 Hospital ward

Perceived stress levels did not differ significantly by nurses’ workplace (ward type).

Ward type incidentally differentiated occupational burnout levels in a statistically significant manner. In each study group, significant differences were observed for only one LBQ dimension. In Slovakia, nurses working in internal medicine wards reported relatively higher burnout, with a statistically significant difference observed only for disappointment (*p* = 0.0206). In Poland, significantly higher psychophysical exhaustion was reported by nurses working in emergency departments (*p* = 0.0364). In contrast, in the Czech sample, nurses working in emergency departments reported lower psychophysical exhaustion (*p* = 0.0102). Detailed results are presented in Table 9.

**Table 9:** Correlations of burnout measures and type of ward.

Ward	Burnout measures (LBQ)			
	Disappointment	Professional effectiveness	Disengagement from patient relationships	Psychophysical exhaustion
Slovakia				
Internal medicine (112)	15.4 (14.2–16.5)	14.6 (13.6–15.5)	16.8 (15.9–17.8)	18.1 (17.1–19.0)
Surgical (65)	13.1 (11.8–14.4)	14.0 (12.8–15.2)	17.7 (16.4–18.9)	18.0 (16.7–19.3)
Emergency (13)	11.9 (8.9–14.9)	11.9 (9.8–14.1)	15.5 (13.0–17.9)	16.3 (13.4–19.2)
*p*	0.0206*	0.1888	0.3038	0.5982
Poland				
Internal medicine (90)	14.1 (12.8–15.4)	13.8 (12.7–14.8)	17.3 (16.0–18.5)	17.1 (15.9–18.4)
Surgical (101)	15.2 (14.0–16.4)	15.0 (13.9–16.2)	17.7 (16.8–18.7)	18.4 (17.3–19.4)
Emergency (53)	15.8 (14.1–17.6)	14.6 (13.3–16.0)	19.2 (17.7–20.7)	19.3 (17.8–20.7)
*p*	0.2032	0.2864	0.0767	0.0364*
Czech rep.				
Internal medicine (78)	16.3 (14.9–17.7)	14.1 (13.1–15.0)	19.9 (18.8–21.1)	21.9 (20.7–23.2)
Surgical (24)	16.1 (13.6–18.6)	14.5 (12.4–16.6)	21.8 (19.3–24.2)	20.3 (17.5–23.1)
Emergency (28)	14.1 (11.5–16.8)	13.7 (11.9–15.5)	18.5 (16.5–20.5)	17.9 (15.5–20.3)
*p*	0.1463	0.7286	0.0970	0.0102*

*p*-test probability values calculated using the Kruskal–Wallis test.

### 3.6 Regression analysis

Multivariable analyses were conducted to assess the combined associations of previously examined factors with occupational burnout. Separate analyses were performed for the Slovak, Polish, and Czech samples. In each group, four regression models were constructed, with individual LBQ burnout dimensions as dependent variables. Variables entered into the multivariable models included demographic and occupational characteristics (age, marital status, education, length of service, ward type) and perceived stress (PSS-10). Stepwise regression was used to identify variables significantly associated with occupational burnout in the multivariable models to obtain parsimonious models with optimal fit. The coefficients of determination (R^2^) varied across models and were generally low to moderate, indicating that the analyzed variables explained only part of the variability in occupational burnout.

#### 3.6.1 Slovak sample

For disappointment, the final model showed significant associations with perceived stress (PSS-10; *p* = 0.0033) and ward type (*p* = 0.0327). An increase of one point in PSS-10 was associated with an average increase of 0.25 points in disappointment. Nurses working in surgical and emergency wards reported lower disappointment compared with those in internal medicine wards, although only the difference between surgical and internal wards was statistically significant (approximately 2.14 points lower). The coefficient of determination was low (*R*^2^ = 9.1%) (Table 10).

**Table 10:** Disappointment, stress intensity, and demographic and occupational factors in the Slovak sample.

Independent variables	Disappointment *R*^2^ = 9.1% *F* = 6.1 *p* = 0.0005***		
	*B* (95% CI)	*p*	*ß*
PSS-10	0.251 (0.085; 0.417)	0.0033**	0.21
Surgical vs. internal medicine	−2.145 (−3.922; −0.367)	0.0183*	−0.17
Emergency vs. internal medicine	−2.747 (−6.104; 0.611)	0.1082	−0.12

*R*^2^ – coefficient of determination, *F* – test statistic and *p*-value for significance of whole model, *B* – regression coefficient with 95% CI, *p*-value for significance of each regression coefficient, *ß* – standardize regression coefficient.

For lack of professional effectiveness, perceived stress was the only variable significantly associated with the outcome (*R*^2^ = 16.6%); each one-point increase in PSS-10 corresponded to a 0.39-point increase in lack of effectiveness. Similarly, for disengagement from patient relationships, perceived stress was the only variable significantly associated with the outcome (*R*^2^ = 7.7%), with a 0.27-point increase per one-point increase in PSS-10. For psychophysical exhaustion, perceived stress was only variable significantly associated with the outcome (*R*^2^ = 15.0%), with an average increase of 0.36 points per one-point increase in PSS-10.

#### 3.6.2 Polish sample

For disappointment, the final model included perceived stress (*p* < 0.001) and length of service (*p* = 0.0118). The model explained 22.8% of variance. A one-point increase in PSS-10 was associated with a 0.36-point increase in disappointment. Nurses with 18–23 years of service reported disappointment levels more than three points higher than those with up to 5 years of service (Table 11).

**Table 11:** Disappointment and the intensity of stress and demographic and professional factors in the Polish sample.

Independent variables		Disappointment *R*^2^ = 22.8% *F* = 11.6 *p* < 0.0001***		
		*B* (95% CI)	*p*	*ß*
PSS-10		0.363 (0.248; 0.478)	<0.0001***	0.37
Length of service	6–11 vs. 0–5 years	2.354 (0.351; 4.356)	0.0214*	0.15
	12–17 vs. 0–5 years	1.141 (−1.177; 3.459)	0.3331	0.06
	18–23 vs. 0–5 years	3.096 (1.172; 5.020)	0.0017**	0.21
	>23 vs. 0–5 years	2.765 (0.543; 4.988)	0.0150*	0.16

*R*^2^ – coefficient of determination, *F* – test statistic and *p*-value for significance of whole model, *B* – regression coefficient with 95% CI, *p*-value for significance of each regression coefficient, *ß* – standardize regression coefficient. *p*-test probability value *p < 0.05, **p < 0.01, ***p < 0.001.

For lack of professional effectiveness, perceived stress was the only variable significantly associated with the outcome, with a relatively high coefficient of determination (*R*^2^ = 26.6%). Each one-point increase in PSS-10 was associated with a 0.36-point increase in lack of effectiveness.

For disengagement from patient relationships, perceived stress and education level were significantly associated with the outcome, with higher engagement observed among nurses with bachelor’s degrees compared with those with secondary education (Table 12).

**Table 12:** Disengagement from patient relationships and the intensity of stress and demographic and professional factors in the Polish sample.

Independent variables	Disengagement from patient relationships *R*^2^ = 25.9% *F* = 20.8 *p* < 0.0001***		
	*B* (95% CI)	*p*	*ß*
PSS-10	0.376 (0.279; 0.474)	<0.0001***	0.44
Bachelor’s degrees vs. secondary	−2.548 (−4.454; −0.642)	0.0090**	−0.23
Master’s degrees vs. secondary	−1.026 (−2.842; 0.789)	0.2666	−0.10

*R*^2^ – coefficient of determination, *F* – test statistic and *p*-value for significance of whole model, *B* – regression coefficient with 95% CI, *p*-value for significance of each regression coefficient, *ß* – standardize regression coefficient.

For psychophysical exhaustion, perceived stress and marital status were significantly associated with the outcome, with the highest exhaustion reported by unmarried nurses (Table 13).

**Table 13:** Psychophysical exhaustion and the intensity of stress and demographic and professional factors in the Polish sample.

Independent variables	Psychophysical exhaustion *R*^2^ = 24.9% *F* = 19.7 *p* < 0.0001***		
	*B* (95% CI)	*p*	*ß*
PSS-10	0.370 (0.269; 0.471)	<0.0001***	0.42
Married vs. partnership	0.798 (−0.649; 2.246)	0.2785	0.07
Single vs. partnership	3.129 (0.779; 5.479)	0.0093**	0.17

*R*^2^ – coefficient of determination, *F* – test statistic and *p*-value for significance of whole model, *B* – regression coefficient with 95% CI, *p*-value for significance of each regression coefficient, *ß* – standardize regression coefficient.

#### 3.6.3 Czech sample

In the Czech sample, regression analyses generally revealed simpler relationships. For disappointment, lack of professional effectiveness, and disengagement from patient relationships, perceived stress was the only variable significantly associated with these burnout dimensions. Coefficients of determination were low, ranging from 5.9% to 16.4%.

For psychophysical exhaustion, a more complex model emerged, including perceived stress (*p* < 0.001), marital status (*p* = 0.0458), education level (*p* = 0.0240), and ward type (*p* = 0.0011). This model demonstrated good fit, explaining nearly 40% of variance. A one-point increase in PSS-10 was associated with a 0.59-point increase in psychophysical exhaustion. Lower exhaustion was observed among nurses working in surgical and emergency wards compared with internal medicine wards (difference exceeding 4.5 points). Nurses with bachelor’s degrees reported lower exhaustion than those with secondary education, and married nurses reported lower exhaustion than those in informal partnerships (Table 14).

**Table 14:** Psychophysical exhaustion and the intensity of stress and demographic and professional factors in the Czech sample.

Independent variables		Psychophysical exhaustion *R*^2^ = 39.5% *F* = 11.0 *p* < 0.0001***		
		*B* (95% CI)	*p*	*ß*
PSS-10		0.593 (0.431; 0.754)	<0.0001***	0.54
Ward	Surgical vs. internal medicine	−2.491 (−4.861; −0.121)	0.0396*	−0.16
	Emergency vs. internal medicine	−4.574 (−6.893; −2.256)	0.0002***	−0.32
Education	Bachelor’s degrees vs. secondary	−3.083 (−5.043; −1.123)	0.0023**	−0.25
	Master’s degrees vs. secondary	−1.935 (−6.174; 2.303)	0.3678	−0.07
Marital status	Married vs. partnership	−2.295 (−4.287; −0.303)	0.0243*	−0.19
	Single vs. partnership	−0.764 (−3.484; 1.956)	0.5791	−0.05

*R*^2^ – coefficient of determination, *F* – test statistic and *p*-value for significance of whole model, *B* – regression coefficient with 95% CI, *p*-value for significance of each regression coefficient, *ß* – standardize regression coefficient.

## 4 Discussion

In this study, psychosocial, demographic, and occupational correlates of occupational burnout were examined among nurses employed in selected hospitals in Central Europe. The findings confirm that burnout in this professional group is a complex phenomenon, significantly associated with perceived stress as well as selected individual and occupational characteristics.

The obtained LBQ scores indicate a moderate level of occupational burnout in the studied sample, with clear differentiation across burnout dimensions. Particularly pronounced were components related to psychophysical exhaustion and disengagement from patient relationships, which are characteristic of care professions involving high emotional demands. This burnout profile is consistent with findings from other studies conducted among nurses, in which the highest levels were observed in exhaustion and emotional distancing (21–23). No statistically significant differences were found between countries for disappointment and reduced professional effectiveness. Significant differences emerged for disengagement and psychophysical exhaustion, with nurses from the Czech sample exhibiting higher mean scores. However, these findings should be interpreted cautiously in light of the lower response rate observed in the Czech sample. The possibility of non-response bias cannot be excluded, particularly if nurses experiencing different levels of burnout differed in their likelihood of participation.

Analysis of perceived stress revealed moderate average PSS-10 scores in the study population; however, a notable finding was that more than one-third of nurses were classified as experiencing high stress. This distribution indicates a substantial psychosocial burden within this professional group. Similar PSS-10 values and proportions of highly stressed nurses have been reported in other hospital-based nursing studies conducted in Europe and beyond (15, 24, 25), reinforcing the notion that occupational stress is a widespread experience in nursing. Nevertheless, the findings should be interpreted with caution because the study relied on a convenience sample and response rates varied between countries. Although the distributions of the main study variables were broadly comparable across the analyzed groups, the possibility of selection and non-response bias cannot be excluded.

One of the key findings of this study is the presence of significant correlations between perceived stress and occupational burnout across all LBQ dimensions. Correlation analyses demonstrated positive associations between PSS-10 scores and burnout dimensions, with Spearman’s rank correlation coefficients ranging from approximately 0.20 to 0.49, indicating weak to moderate relationships. The strongest associations were observed for reduced professional effectiveness and psychophysical exhaustion, suggesting that higher perceived stress co-occurs with increased mental burden and diminished occupational functioning. These findings are consistent with previous research documenting moderate to strong associations between stress and burnout among nurses across different organizational contexts (6, 11, 21). Given the cross-sectional design, these relationships should be interpreted as co-occurring rather than causal.

Demographic correlates of stress were limited. Only one statistically significant, very weak negative correlation between age and stress was observed in the Czech sample, indicating slightly lower stress levels among older nurses. This pattern aligns with previous findings suggesting that younger or less experienced nurses may be more vulnerable to psychosocial strain (11, 26). Family-related variables generally did not differentiate stress levels, suggesting that occupational rather than domestic factors may play a more prominent role in perceived stress, as reported elsewhere (27, 28).

Occupational variables such as education level, ward type, and length of service showed little association with stress levels, which is consistent with studies emphasizing the primacy of organizational and psychosocial demands over clinical specialization in shaping stress experiences (29, 30). However, this contrasts with research suggesting that greater experience may buffer stress through enhanced coping strategies (30, 31).

Analysis of demographic correlates indicated that selected individual characteristics of nurses were statistically significantly associated with occupational burnout; however, the strength of these associations was generally weak. These findings are consistent with previous studies suggesting that demographic factors such as age or family situation play a secondary role in burnout compared with psychosocial and organizational work-related burdens (8, 32). The associations observed in the Czech sample across age, having children, and length of service should be interpreted with caution. Although some statistically significant results were identified, they were weak in magnitude and not consistently observed across related variables. In combination with the lower response rate in this cohort, these findings may be influenced by increased uncertainty and potential non-response bias, and should therefore be interpreted as exploratory rather than confirmatory.

However, it should be noted that the literature is not fully consistent regarding the role of demographic factors in burnout. Some studies have reported higher levels of burnout among older nurses and among women with greater family responsibilities, including marital status and having children, particularly in mid- to late-career stages, although findings across studies remain heterogeneous and context-dependent (6). Similarly, evidence suggests that burnout may vary across different stages of professional development, with both early-career nurses (due to limited experience and coping resources) and more experienced staff (due to cumulative occupational burden) being at increased risk (33). In addition, the role of family-related factors such as marital status and having children appears inconsistent across studies, with some evidence suggesting increased strain due to competing work–family demands, while other studies do not confirm a significant association (34). Overall, these findings indicate that the relationship between demographic characteristics and burnout is likely to be complex and influenced by both occupational and contextual factors.

The fact that burnout levels were rarely and only weakly associated with having children or marital status may be interpreted as a favorable finding. It suggests that nurses do not strongly link their occupational functioning with their domestic situation and that burnout is not primarily rooted in family-related responsibilities. Similarly, the absence of meaningful correlations—or the presence of only negligible associations—between age and LBQ dimensions indicates that occupational burnout is not an inevitable consequence of increasing age or length of professional experience. Rather, burnout appears to be a condition that may affect nurses at various stages of their careers, depending on work-related conditions. From a public health practice perspective, these observations highlight the need to focus preventive efforts primarily on workplace conditions and occupational stress rather than on individual demographic characteristics of healthcare workers.

Equally important were the associations observed between occupational burnout and selected occupational factors, such as length of service and characteristics of the work environment. Although these relationships were relatively infrequent and generally weak, their presence is consistent with conceptual frameworks suggesting that prolonged exposure to psychosocial demands in the workplace may co-occur with higher levels of burnout (23, 35). From a public health perspective, these findings underscore the importance of organizational factors as potential targets for interventions aimed at improving nurses’ well-being.

Multivariable analysis constituted a key component of the present study and directly addressed one of the predefined research questions concerning the identification of factors independently associated with the severity of occupational burnout among nurses. The use of multivariable models allowed for the assessment of the relative importance of perceived stress, demographic characteristics, and occupational factors while simultaneously controlling for their co-occurrence, which is particularly important in research on complex psychosocial phenomena.

Across all models, perceived stress (PSS-10) remained a statistically significant and the strongest correlate of occupational burnout in all dimensions measured by the LBQ. This finding is consistent with previous studies in which stress has consistently been identified as one of the factors most strongly associated with burnout in medical professions, even after accounting for other individual and organizational variables (36, 37). These authors emphasize that occupational stress often functions as a “common denominator” underlying multiple work-related burdens in nursing practice.

At the same time, in the multivariable models, most demographic variables—such as age and marital status—did not demonstrate independent associations with burnout severity. This suggests that their role may be secondary to psychosocial factors. Similar conclusions have been reported in population-based studies showing that, after controlling for stress, social support, and work-related demands, demographic characteristics tend to lose statistical significance (38, 39). These findings indicate that individual characteristics alone are insufficient to explain variability in burnout levels.

Occupational factors, including length of service and characteristics of the work environment, showed relatively weak independent associations with occupational burnout in the multivariable models. These results are consistent with studies emphasizing that tenure or ward type tends to be relevant primarily in interaction with psychosocial workload and organizational climate rather than as independently associated with burnout (40, 41). It should also be noted that several regression models demonstrated relatively low coefficients of determination (R^2^), indicating that a substantial proportion of variability in occupational burnout remained unexplained. This suggests that occupational burnout is a multifactorial phenomenon likely influenced by additional organizational, systemic, and psychosocial factors not included in the present study, such as workload, staffing levels, leadership style, organizational climate, social support, or coping strategies.

At the same time, given the complexity and multidimensional nature of burnout, relatively low R^2^ values are not uncommon in psychosocial research and do not negate the relevance of the observed group-level associations. Nevertheless, despite limited precision of individual-level estimates, the identified associations may still provide meaningful information at the group or workplace level and may support the identification of work environments characterized by higher average burnout burden.

From a public health perspective, the results of the multivariable analysis highlight that occupational burnout among nurses is a multidimensional phenomenon in which subjectively perceived stress plays a central role, rather than isolated demographic or occupational characteristics. This perspective aligns with job demands–resources and workload models, which emphasize the importance of the work environment and psychosocial working conditions for the health of medical personnel (32, 42, 43).

Despite several limitations, the study makes an important contribution to the literature by providing data on correlates of occupational burnout in a Central European context, a region that remains relatively underrepresented in international research. The present study is based on a multi-site, hospital-based sample drawn from three Central European countries and analyzed using a harmonized methodological framework. Importantly, it was not designed to support direct country-level comparisons, but rather to capture variability across clinical settings within a broader regional context.

The study further extends current knowledge by conceptualizing occupational burnout as a multidimensional construct assessed with the Link Burnout Questionnaire, which enables a more differentiated analysis of specific burnout components, including emotional disengagement and psychophysical exhaustion. In addition, the findings from multivariable regression models demonstrate a consistent and predominant association between perceived stress and all burnout dimensions compared with demographic and occupational variables, thereby reinforcing the robustness of this relationship across different hospital settings.

From a public health perspective, the results underscore the need for systematic monitoring of stress and occupational burnout among nurses, as well as the implementation of interventions aimed at improving working conditions and reducing psychosocial burdens in healthcare environments. At the same time, the presented findings should be interpreted in the context of the relatively narrow operationalization of psychosocial factors, which in this study focused primarily on perceived stress. Other relevant psychosocial and organizational determinants, including social support, leadership style, organizational climate, staffing levels, and coping strategies, were not assessed and may also substantially contribute to burnout severity.

Future research should incorporate longitudinal designs to better understand the dynamics of occupational burnout and the temporal relationships between stress and other psychosocial factors, which may provide valuable insights for the development of effective preventive and intervention strategies in nurses’ occupational health.

## 5 Conclusion

1. Occupational burnout among nurses employed in selected hospitals in Central European countries is significantly associated with perceived stress levels.
2. Demographic and occupational factors show generally weaker independent associations with burnout than perceived stress, highlighting the need to focus interventions on psychosocial work-related burdens rather than individual characteristics.
3. Multivariable regression models confirmed perceived stress as the strongest and most stable correlate of occupational burnout, retaining its significance after adjustment for demographic and occupational variables.
4. Identifying factors co-occurring with occupational burnout may inform the development of targeted interventions aimed at improving working conditions and supporting nurses’ psychological well-being.

## 6 Methodological limitations

Several methodological limitations should be considered when interpreting the results of this study:

1. The cross-sectional design does not allow for causal inferences; therefore, the observed relationships between perceived stress, occupational burnout, and demographic or occupational variables should be interpreted as associations rather than causal or predictive relationships.
2. The study relied on a convenience sampling strategy, as participation depended on nurses’ availability during the data collection period and their willingness to participate. Consequently, the study sample may not fully represent the broader population of hospital nurses, which introduces a risk of selection bias. Nurses experiencing particularly high or particularly low levels of burnout may have differed in their likelihood of participation, potentially leading to over- or underestimation of the observed associations.
3. Variability in response rates across countries—particularly the lower response rate observed in the Czech sample—may have introduced non-response bias. Because the study was conducted anonymously, no data were collected on non-respondents, and it was therefore not possible to assess whether respondents differed systematically from nurses who did not participate. Consequently, findings related to the Czech cohort should be interpreted with caution.
4. Participants were recruited from selected hospitals in Central European countries, which limits the generalizability of the findings. Therefore, the results should not be interpreted as representative of national nursing populations or healthcare systems.
5. The study was based on self-reported measures of perceived stress (PSS-10) and occupational burnout (LBQ). Although both instruments are validated and widely used, self-report data may be affected by response bias, including social desirability and subjective perceptions of occupational experiences.
6. Although validated Polish versions of the instruments served as the basis for the study, the Czech and Slovak versions were developed through translation and linguistic harmonization without a full formal cross-cultural adaptation procedure. Although efforts were made to ensure conceptual consistency across language versions, the absence of procedures such as forward–backward translation, independent translation verification, and formal equivalence testing may have affected measurement invariance and the comparability of results between countries.
7. Internal consistency of the instruments was confirmed in the study sample; however, structural validity and measurement invariance across language versions were not formally tested. In accordance with COSMIN recommendations, this limits the strength of conclusions regarding cross-cultural comparability of the burnout construct.
8. While the Link Burnout Questionnaire (LBQ) is a validated instrument with documented psychometric properties, it is less frequently used internationally than more widely established measures such as the Maslach Burnout Inventory, which may limit direct comparability of the findings with other studies conducted in different settings.
9. Despite the inclusion of several demographic and occupational variables, other potentially important organizational and psychosocial factors, such as workload indicators, staffing levels, organizational climate, leadership style, social support, or coping strategies, were not assessed and may have contributed substantially to burnout variability and to the relatively low proportions of variance accounted for by some regression models.

## Data Availability

The raw data supporting the conclusions of this article will be made available by the authors, without undue reservation.
